# Motion Clutter Suppression for Non-Cooperative Target Identification Based on Frequency Correlation Dual-SVD Reconstruction

**DOI:** 10.3390/s24165298

**Published:** 2024-08-15

**Authors:** Weikun He, Yichuan Luo, Xiaoxiao Shang

**Affiliations:** Tianjin Key Lab for Advanced Signal Processing, Civil Aviation University of China, Tianjin 300300, China; luoyichuan1234567@163.com (Y.L.); xxshang512@163.com (X.S.)

**Keywords:** target identification of birds and UAVs, strong motion clutter, frequency correlation dual-SVD reconstruction, clutter suppression, micro-motion characteristics

## Abstract

Non-cooperative targets, such as birds and unmanned aerial vehicles (UAVs), are typical low-altitude, slow, and small (LSS) targets with low observability. Radar observations in such scenarios are often complicated by strong motion clutter originating from sources like airplanes and cars. Hence, distinguishing between birds and UAVs in environments with strong motion clutter is crucial for improving target monitoring performance and ensuring flight safety. To address the impact of strong motion clutter on discriminating between UAVs and birds, we propose a frequency correlation dual-SVD (singular value decomposition) reconstruction method. This method exploits the strong power and spectral correlation characteristics of motion clutter, contrasted with the weak scattering characteristics of bird and UAV targets, to effectively suppress clutter. Unlike traditional clutter suppression methods based on SVD, our method avoids residual clutter or target loss while preserving the micro-motion characteristics of the targets. Based on the distinct micro-motion characteristics of birds and UAVs, we extract two key features: the sum of normalized large eigenvalues of the target’s micro-motion component and the energy entropy of the time–frequency spectrum of the radar echoes. Subsequently, the kernel fuzzy c-means algorithm is applied to classify bird and UAV targets. The effectiveness of our proposed method is validated through results using both simulation and experimental data.

## 1. Introduction

The detection of low-altitude targets by radar is often compromised by strong motion clutter from sources such as airplanes (in apron environments) and cars (on a highway). This interference makes it challenging to distinguish between birds and UAVs, which possess weak scattering characteristics and can easily be obscured by clutter. Therefore, developing effective clutter suppression methods for the identification of birds and UAVs in environments with strong motion clutter is crucial for enhancing the performance of radar detection systems [1,2].

Regarding strong clutter suppression, scholars have conducted extensive research focusing on methods such as adaptive moving target indication (AMTI) [3], the CLEAN algorithm [4], wavelet transform techniques [5,6,7,8,9,10], and singular value decomposition (SVD) [11,12,13,14,15]. Among these, SVD-based motion clutter suppression methods have garnered significant attention. Zheng Lin et al. [11] proposed a clutter suppression method that combines phase encoding with SVD. This method first whitens the clutter through phase decoding and then separates the clutter based on eigenvalue differences in the autocorrelation matrix between the whitened clutter and the target. Additionally, the wavelet-SVD algorithm involves performing a two-dimensional wavelet transform and SVD on the transformed wavelet coefficient matrix, followed by the removal of larger singular values to suppress clutter [12]. M. Garcia Fernandez et al. [13] introduced a clutter suppression method for synthetic aperture radar (SAR) images. In this method, clutter and target subspaces are constructed through the SVD of SAR images, and the bases corresponding to clutter are selected. Radar echoes are then projected onto the orthogonal complement of the clutter subspaces to achieve clutter suppression. Reference [14] utilizes three statistical features: singular value spectral distribution, singular vector space correlation (where ground clutter in different range resolution cells exhibits strong correlation), and average Doppler frequency. These features are used to adaptively determine the singular vectors corresponding to the clutter subspace via the K-means clustering algorithm, and clutter components are subsequently suppressed through orthogonal subspace projection. Wu Linyong et al. [15] proposed a first-order differential spectral SVD reconstruction method. This technique employs the first-order differential spectrum of singular values to classify and reconstruct radar echoes, effectively distinguishing between clutter and targets. Cui Weicheng et al. [16] proposed a method for selecting singular values based on the principle of minimizing the fitting error.

In the realm of target identification methods for UAVs and birds, there are two primary approaches: those based on feature extraction and those that combine feature extraction with machine learning. Key extracted features include spectral symmetry pairs of radar echoes [17], motion model conversion frequency [18], the first left singular vector corresponding to singular value decomposition, and the cadence velocity diagram (CVD) [19], as well as target velocity, spectral period, and width [20]. Additionally, features such as the mean and variance of singular values are also utilized [21]. Identification methods that integrate feature extraction with machine learning include various combinations of feature vectors and classifiers. For instance, methods include using a feature vector corresponding to the first two largest eigenvalues combined with a support vector machine (SVM) [22], a micro-Doppler bandwidth combined with a K-nearest neighbor (KNN) classifier [23], Doppler images derived from micro-Doppler signatures (MDS) and rhythmic velocimetry maps combined with a convolutional neural network (CNN) [24], and synthetic micro-Doppler spectrograms combined with a CNN [25]. Reference [26] compares the classification effectiveness of CNNs and autoencoders. Beom Seok Oh et al. [27] designed an automatic recognition and classification system. This system employs empirical mode decomposition (EMD) to decompose and reconstruct radar echoes from UAVs, birds, humans, vehicles, and other objects. Signal energy, logarithmic energy entropy, and spectral energy density are then extracted to classify UAVs, birds, pedestrians, and vehicles using SVM. Additionally, wavelet decomposition is applied to radar echoes of UAVs and robotic birds, allowing for the estimation of bird flapping frequencies and drone rotor speeds for target identification [28].

**Figure 1:**
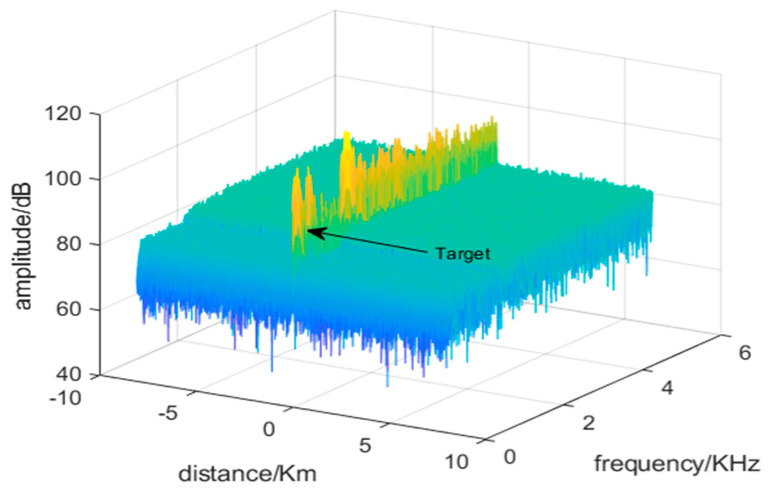
Range-Doppler spectrum with strong motion clutter interference.

The previously discussed methods for identifying birds and UAVs do not address the interference caused by strong motion clutter in real-world observation environments (as shown in Figure 1, the target is obscured by strong clutter). As a result, detecting the target within this clutter becomes challenging.

Moreover, SVD-based clutter suppression methods encounter several challenges. First, selecting the appropriate singular values is difficult. Second, in practical scenarios, the intermingling of clutter and target information can cause the singular vectors corresponding to these singular values to contain both, potentially leading to the loss of target signals.

The frequency correlation dual-SVD reconstruction method is proposed to address the impact of strong motion clutter on the discrimination performance of birds and UAVs. This method aims to remove motion clutter while preserving the micro-motion information of targets. Compared to first-order differential spectrum SVD reconstruction (FODS-SVD) in reference [15] and fitting error minimum principle SVD reconstruction (FEMP-SVD) in reference [16], the main differences in the proposed method lie in the selection of singular values and the handling of clutter intertwined with target information. The FODS-SVD and FEMP-SVD method only analyze how to select singular values and do not consider the intermingling of target and clutter information. Additionally, they do not account for the possibility that, after singular value decomposition, the singular values representing clutter components may be smaller than those representing target information (leading to residual clutter). The proposed method has already solved the two issues mentioned above. The target identification section is primarily used to verify the effectiveness of this method.

The content of this paper is arranged as follows. The second section primarily discusses the innovative clutter suppression method proposed in this study. The third section focuses on extracting the features of birds and UAVs and implementing their identification using the kernel fuzzy c-means (KFCM) method. The fourth section analyzes the results based on both simulated and experimental data. The fifth section provides a summary of the content of this paper.

## 2. Frequency Correlation Dual-SVD Reconstruction Based Clutter Suppression

### 2.1. General Approach to Clutter Suppression

Before discriminating between non-cooperative targets, ground clutter is suppressed using moving target detection (MTD) [29]. Initially, the constant false alarm rate (CFAR) [30] technique is employed to detect strong motion clutter and suppress it using the proposed method. Next, the micro-motion characteristics of the targets are assessed. Only range resolution cells exhibiting these characteristics are retained, while cells containing only clutter are removed. Consequently, subsequent processing focuses on the range resolution cells where both targets and clutter coexist.

In the proposed method, the strong power and spectral correlation of the motion clutter are utilized, and the received signal is processed using the corresponding Hankel matrix [16]. The spectral correlation of the strong motion clutter is transformed into the spectral correlation of the singular vectors obtained through singular value decomposition (SVD) of the Hankel matrix corresponding to the radar echoes. Since the power of the motion clutter significantly exceeds that of the target [31], singular vectors mainly containing clutter components can be identified based on the correlation between the spectrum of each singular vector and that of the singular vector with the maximum singular value.

**Figure 2:**
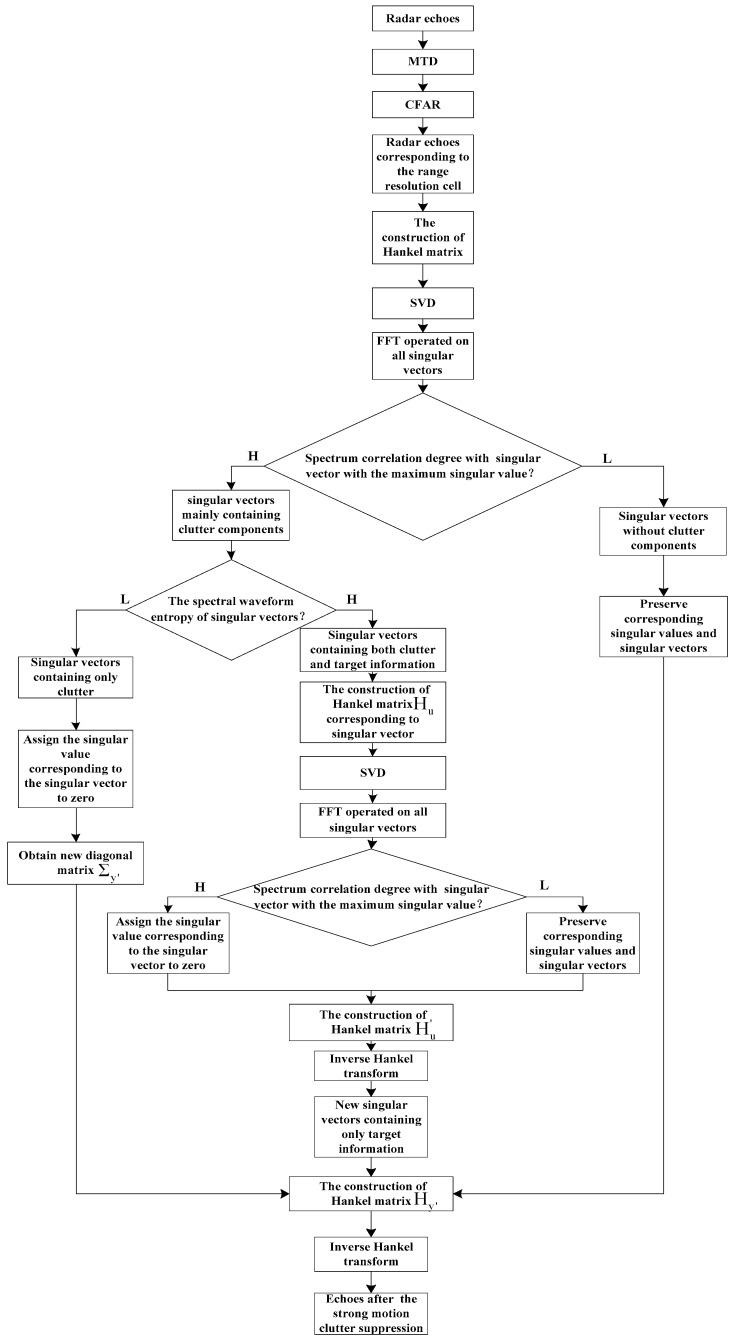
The block diagram of the method based on frequency correlation dual SVD reconstruction.

To preserve target micro-motion information, spectral waveform entropy is used as a quantitative evaluation index for extracting singular vectors containing both clutter and target information. This spectral correlation characteristic is then utilized again to suppress the strong motion clutter component in singular vectors containing both clutter and target information. Then, new singular vectors containing only target information can be obtained. Finally, by selecting different singular values and singular vectors corresponding to the target and clutter, the new Hankel matrix is reconstructed. The inverse Hankel transform is subsequently applied to obtain the target components. A block diagram of the proposed method is shown in Figure 2.

### 2.2. Determination of the Singular Values and Singular Vectors Corresponding to the Clutters

The received echoes corresponding to the range resolution cell of interest are the sequence of sampled instantaneous amplitudes of the received electromagnetic signal over the sampling periods, which can be represented as

(1) X=s+c+n

where s is the bird or UAV signal, c is strong motion clutter such as a car or plane, and n is noise. The clutter components exhibit spectral correlation due to the similarity in the spectral shape and center frequency of the motion clutter components. To utilize the spectral correlation of strong motion clutter components, the received signal X=[x(1),x(2),⋯,x(N)] is first converted into a two-dimensional Hankel matrix $H_{x}$, which can be written as follows:

(2) $$H_{x}=\left[\begin{array}{cccc} x(1) & x(2)\; & \cdots & \;x(J)\\ x(2) & x(3)\; & \cdots & \;x(J+1)\\ \vdots & \vdots & & \vdots \\ x(M) & x(M+1)\; & \cdots & \;x(N) \end{array}\right]$$

where N=M+J−1 is the received signal length, M=(N+1)/2 when N is an odd number, and M=N/2 when N is even. J can be obtained by J=N+1−M. It can be observed that each row of the Hankel matrix $H_{x}$ has J-1 elements that are the same as the previous row, indicating a correlation between the rows. $H_{x}$ is decomposed by the SVD as follows:

(3) $$H_{x}{=U}_{x}\Sigma_{x}V_{x}^{H}$$

The left singular matrix $U_{x}{=[u}_{1}{,u}_{2},\cdots {,u}_{M}]\in C^{M\times M}$ and the right singular matrix $V_{x}{=[v}_{1}^{H}{,v}_{2}^{H},\cdots {,v}_{J}^{H}]\in C^{J\times J}$ are the orthogonal matrices, $\Sigma_{x}\in C^{M\times J}$ is the diagonal matrix composed of the corresponding singular values in descending order, namely

(4) $$\Sigma_{x}=\left\{ \begin{array}{ll} {\;\mathrm{diag}(\sigma }_{1}{,\sigma }_{2},\cdots {,\sigma }_{r}) & M=J\\ {\;[\mathrm{diag}(\sigma }_{1}{,\sigma }_{2},\cdots {,\sigma }_{r}{),O]}^{T} & M\text{<}J \end{array}\right.$$

where r=min(M,J) represents the number of singular values, and O represents the zero matrix. The received signal information is contained in the obtained singular values and singular vectors. Therefore, the essence of the proposed clutter suppression method is to eliminate strong motion clutter components in all singular vectors.

The left and right singular vectors in Equation (3) represent the time-domain characteristics of the received signal. To utilize the spectral correlation of the motion clutter components, the fast Fourier transform (FFT) is applied to each of the left singular vectors, as shown below:

(5) $${\overset{¯}{U}}_{i}{(k)=\mathrm{FFT}[u}_{i}(n)]=\sum_{n=0}^{M-1}u_{i}{(n)W}_{M}^{\mathrm{kn}}=\sum_{n=0}^{M-1}u_{i}\left(n\right)e^{-j2\pi kn/M},\;k=0,1,2,\cdots ,M-1$$

Since each element on the anti-diagonals of the Hankel matrix $H_{x}$ is equal and the left and right singular vectors are orthogonal to each other, the spectra of the left and right singular vectors are symmetric about zero frequency [32], and thus only the correlation between the spectra of the left singular vectors needs to be analyzed. Considering that the clutter power significantly exceeds that of the target, the left singular vector corresponding to the maximum singular value is regarded as the clutter component. Therefore, it is necessary to calculate the Pearson correlation coefficients [33] between the spectra of all singular vectors and the spectrum of the left singular vector corresponding to the maximum singular value. Singular vectors with higher Pearson correlation coefficients are extracted because they predominantly contain clutter components. This approach prevents the issue where singular values representing clutter are smaller than those representing target information, which could lead to residual clutter.

To preserve the micro-motion features of the target while suppressing clutter, the singular vectors mainly containing clutter components are categorized into those containing only clutter and those containing both clutter and target information. Given the complexity and randomness of the spectral distribution of singular vectors, spectral waveform entropy [34] is introduced. Taking any spectral sequence $\overset{¯}{U}\left(k\right)$ as an example, the probability distribution P(k) of the frequency components is calculated, namely

(6) $$P(k)={\left|\overset{¯}{U}\left(k\right)\right|}^{2}/\sum_{k=0}^{M-1}\left({\left|\overset{¯}{U}\left(k\right)\right|}^{2}\right)\;$$

The corresponding spectral waveform entropy can be expressed as follows:

(7) $$Q=-\sum_{k=0}^{M-1}P(k)\log(P(k))\;$$

That is, the larger the spectral waveform entropy value, the more frequency components are included. A higher entropy value of the spectral waveform is indicative of a singular vector containing both target and clutter information, while a lower entropy value suggests a singular vector containing only clutter. The singular values corresponding to the singular vectors containing only clutter are set to zero, resulting in a new diagonal matrix $\Sigma_{y\prime }$. However, the left or right singular vectors ($u_{c+t}$ or $v_{c+t}$) containing both clutter and target information require further processing.

SVD reconstruction is performed again to suppress the motion clutter for the singular vectors containing both clutter and target information. Unlike the initial SVD reconstruction, the Hankel matrix $H_{u}$ is constructed from each left singular vector containing clutter and target information., specifically:

(8) $$H_{u}=\left[\begin{array}{ccccc} u_{c+t}\left(1\right) & u_{c+t}(2)\; & \cdots & u_{c+t}\left(L\right)\\ u_{c+t}(2) & u_{c+t}(3)\; & \cdots & {\;u}_{c+t}\left(L+1\right)\\ \vdots & \vdots & & \vdots & \\ u_{c+t}(Z) & u_{c+t}(Z+1)\; & \cdots & {\;u}_{c+t}\left(M\right) \end{array}\right]$$

Z=(M+1)/2 when M is an odd number, and Z=M/2 when M is even, and L can be obtained by L=M+1−Z. $H_{u}$ is subjected to SVD, and $U_{u}$, $\Sigma_{u}$, and $V_{u}^{H}$ are obtained. Then, the Pearson correlation coefficients between the spectrum of the left singular vector and that of the left singular vector corresponding to the maximum singular value are calculated. The singular values corresponding to singular vectors with relatively larger Pearson correlation coefficient values are set to zero. The new diagonal matrix $\Sigma_{u}^{\prime }$ is obtained. The Hankel matrix $H_{u}^{\prime }$ is then reconstructed using $U_{u}$, $\Sigma_{u}^{\prime }$, and $V_{u}^{H}$, specifically:

(9) $$H_{u}^{\prime }{=U}_{u}\Sigma_{u}^{\prime }V_{u}^{H}=\left[\begin{array}{cccc} u_{\mathrm{tar}}\left(1\right) & u_{\mathrm{tar}}(2)\; & \cdots & {\;u}_{\mathrm{tar}}\left(L\right)\\ u_{\mathrm{tar}}(2) & u_{\mathrm{tar}}(3) & \cdots & u_{\mathrm{tar}}\left(L+1\right)\\ \vdots & \vdots & & \vdots \\ u_{\mathrm{tar}}(Z) & u_{\mathrm{tar}}\left(Z+1\right) & \cdots & u_{\mathrm{tar}}\left(M\right) \end{array}\right]$$

The inverse Hankel transform on $H_{u}^{\prime }$ can also be used to obtain new singular vectors containing only target information, which can then replace the original singular vector containing both clutter and target information. The new left and right singular matrices, $U_{y\prime }$ and $V_{y\prime }^{H}$, are obtained.

On this basis, the diagonal matrices $\Sigma_{y\prime }$, along with the left and right singular matrices $U_{y\prime }$ and $V_{y\prime }^{H}$, are then used to reconstruct the new Hankel matrix $H_{y\prime }$, specifically:

(10) $$H_{y\prime }{=U}_{y\prime }\Sigma_{y\prime }V_{y\prime }^{H}=\left[\begin{array}{cccccc} y\prime \left(1,1\right) & \;y\prime (1,2)\; & \cdots & \;y\prime (1,j)\; & \cdots & \;y\prime \left(1,J\right)\\ y\prime (2,1) & y\prime (2,2)\; & \cdots & \;y\prime (2,j)\; & \cdots & \;y\prime \left(2,J\right)\\ \vdots & \vdots & & \vdots & & \vdots \\ y\prime \left(m,1\right) & \;y\prime (m,2)\; & \cdots & \;y\prime (m,j)\; & \cdots & \;y\prime (m,J)\;\\ \vdots & \vdots & \vdots & \vdots \\ y\prime (M,1) & y\prime (M,2)\; & \cdots & \;y\prime (M,j)\; & \cdots & \;y\prime \left(M,J\right) \end{array}\right]$$

where y′(m,j) represents the element in the reconstructed Hankel matrix $H_{y\prime }$. Then, $H_{y\prime }$ is subjected to an inverse Hankel transformation, which is shown as follows

(11) $$\left\{ \begin{array}{l} y(1)=y\prime \left(1,1\right)\\ y(2)=[y\prime (1,2)+y\prime (2,1)]/2\\ \;\vdots \\ y(N-1)=[y\prime (M-1,J)+y\prime (M,J-1)]/2\\ y(N)=y\prime \left(M,J\right) \end{array}\right.$$

The signal after clutter suppression can be written as follows:

(12) Y=[y(1),y(2),⋯,y(N)]

## 3. The Kernel Fuzzy C-Means-Based Identification of Birds and UAVs

**Figure 3:**
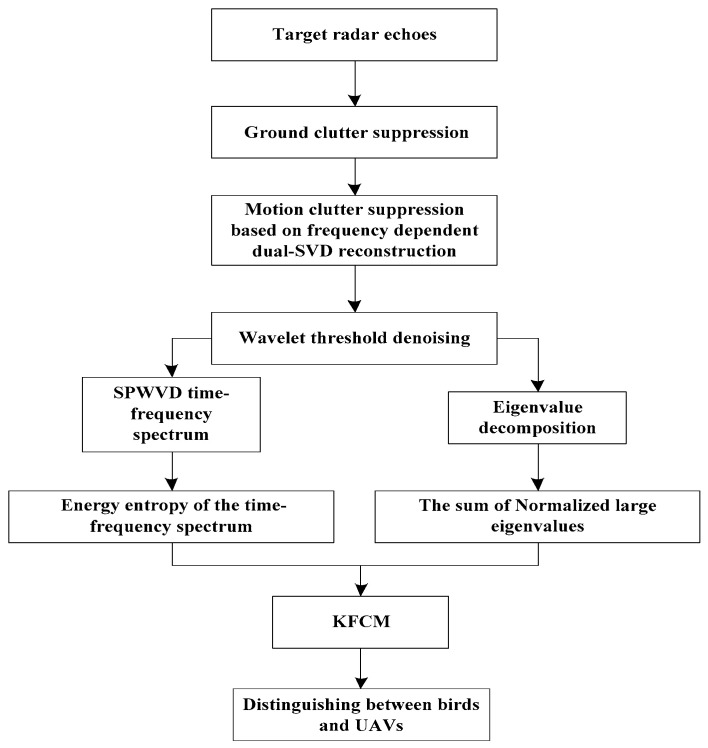
Block diagram of the bird and UAV targets discrimination against the background of strong motion clutter.

After suppressing strong moving clutter, considering that energy entropy is easily affected by noise, the wavelet threshold denoising (WTD) method is used to denoise the radar echoes. In this method, the wavelet coefficients corresponding to the signal components are larger, while those corresponding to the noise components are much smaller. By selecting an appropriate threshold, the wavelet coefficients below this threshold are set to zero, and wavelet reconstruction is performed to obtain the denoised signal $Y_{\mathrm{denoised}}$. Feature extraction is then carried out, and the identification of birds and UAVs is achieved using the KFCM method. The flowchart for identifying birds and UAVs is shown below. See Figure 3.

### 3.1. Feature Extraction

#### 3.1.1. Energy Entropy of the Time–Frequency Spectrum

A time–frequency spectrum is used to depict the variance of the frequency distribution with time for a signal. The time–frequency spectrum can simultaneously display the variations of a signal in both the time and frequency domains, making it particularly suitable for analyzing non-stationary signals that change over time. Due to the flapping wings of birds, the time–frequency spectrum is less orderly compared to that of UAVs, which exhibit strong periodicity. According to the physical interpretation of entropy, a larger entropy value indicates a more disordered state. Therefore, the energy entropy of the time–frequency spectrum of the received radar echoes can be extracted as a micro-motion feature to distinguish between birds and UAVs.

The smoothed pseudo-Wigner–Ville distribution (SPWVD) effectively reduces cross-term interference and exhibits better energy concentration through windowing and smoothing in both the time and frequency domains. Therefore, SPWVD is selected for time–frequency analysis, as shown in Equation (13).

(13) $$G(t,f)=\sum_{u=-\infty }^{+\infty }\sum_{\tau =-\infty }^{+\infty }{h(\tau )g(u)Y}_{\mathrm{denoised}}(t-u+\frac{\tau }{2}{)Y}_{\mathrm{denoised}}^{*}(t-u-\frac{\tau }{2}{)e}^{j2\pi f\tau }$$

By performing singular value decomposition on G(t,f), the resulting left singular matrix $U_{g}$ represents the frequency domain information. The time–frequency spectrum energy entropy η is then defined as follows:

(14) $$\eta =-\sum_{n=1}^{N}q_{n}{\mathrm{lnq}}_{n}$$

where $q_{n}{=E}_{n}/\left(\sum_{n=1}^{N}E_{n}\right)$, $E_{n}={\left|{\left(U_{g}\right)}_{\left(n,1\right)}\right|}^{2}+{\left|{\left(U_{g}\right)}_{\left(n,2\right)}\right|}^{2}$, ${\left|{\left(U_{g}\right)}_{\left(n,1\right)}\right|}^{2}$ and ${\left|{\left(U_{g}\right)}_{\left(n,2\right)}\right|}^{2}$, respectively, represent the energy of the first and second left singular vectors for the n-th sample, and $\sigma_{n}^{\prime }$ represents the n-th eigenvalue. The aforementioned entropy can be used to preliminarily distinguish between birds and UAVs.

#### 3.1.2. Sum of the Normalized Large Eigenvalues

Due to the higher rotational speed of a UAV’s rotor, the spectrum of the UAV exhibits a wider bandwidth and smaller spacing between spectral lines compared to that of birds. Therefore, the sum of the normalized larger eigenvalues for the autocorrelation matrix of the target echoes is used to characterize the spectral width and the number of spectral lines for the target.

By exploiting the distinct sparse characteristics of the micro-motion components (flapping wing components for birds and rotor components for UAVs) and the body, the micro-motion components $y_{w}$ of the signals $Y_{\mathrm{denoised}}$ are first extracted using the augmented Lagrangian method. The autocorrelation matrix of $y_{w}$ can be represented as follows:

(15) $$R=\frac{1}{N}y_{w}y_{w}^{H}$$

where ${\left(\text{•}\right)}^{H}$ represents a complex conjugate. The matrix R is subjected to eigenvalue decomposition (EVD), specifically:

(16) $${R=U}_{r}\Sigma_{r}U_{r}^{H}$$

$\Sigma_{r}{=\mathrm{diag}[\lambda }_{1}{,\lambda }_{2},\cdots {,\lambda }_{N}]$. $\lambda_{1}\geq \lambda_{2}\geq \cdots \geq \lambda_{N}$ represents the diagonal matrix composed of the corresponding eigenvalues. The sum of normalized larger eigenvalues is defined as follows:

(17) $$f_{Z}=\sum_{i=1}^{N_{c}}\lambda_{i}{/\lambda }_{1}$$

where $N_{c}$ is the number of the larger eigenvalues, which are defined as those greater than one percent of the maximum eigenvalue [35]. Compared to the feature spectrum of birds, the spectrum of the UAVs has a greater number of larger eigenvalues and exhibits relatively stable changes. Therefore, the sum of normalized large eigenvalues for birds is generally smaller than that for UAV targets.

### 3.2. The Kernel Fuzzy C-Means Method for Distinguishing Birds and UAVs

Traditional hard clustering algorithms exhibit poor classification performance due to their strict membership degree of either zero or one. In contrast, fuzzy clustering algorithms extend the membership degree to any value between 0 and 1. This flexibility is not limited by the shape of the samples and can greatly enhance the identification performance of fuzzy datasets. The kernel fuzzy c-means (KFCM) clustering algorithm is an improved version of the traditional fuzzy c-means clustering algorithm. By introducing kernel functions, data can be projected into higher-dimensional feature spaces, facilitating easier separation of the data in these spaces. This approach improves both the discrimination accuracy and the stability of target identification.

For the i-th ($i=1,2,\cdots {,N}_{s}$, where $N_{s}$ represents the number of samples) given target echo sequence of length N, we calculate a single value $\eta_{i}$ of the time–frequency spectrum and a single value $f_{Z_{i}}$ representing the sum of normalized largest eigenvalues, and then combine them into a point $a_{i}$ as follows:

(18) $$a_{i}{=(\eta }_{i}{\;f}_{Z_{i}})$$

In the experiment, N target echoes will generate N feature points. Then, by clustering these feature points using the KFCM method, the distinction between birds and drones can be achieved.

## 4. Results and Analysis

### 4.1. Results and Analysis of the Simulation Data

In the simulation experiment, an S-band linear frequency modulation pulse radar system was adopted. The signal-to-clutter ratio (SCR) corresponding to the motion clutter was −25 dB. The parameters of the radar, UAVs, and birds are shown in Table 1, Table 2 and Table 3. The coherent processing interval (CPI) was 256 milliseconds, so the signal length N was 3200. The spectral correlation threshold was set to 0.8 and the spectral entropy threshold was set to 7 bits in the clutter suppression method.

**Figure 4:**
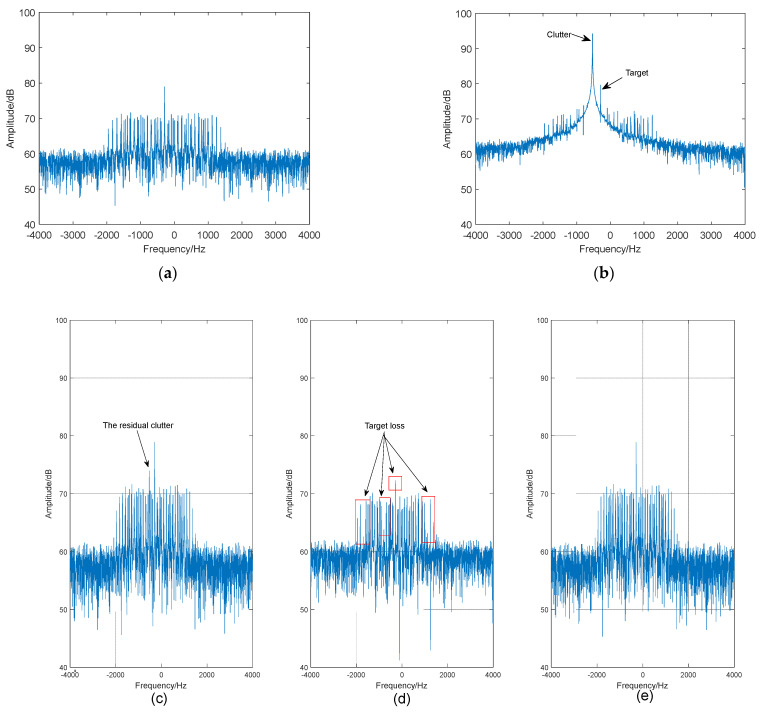
Clutter suppression results of a UAV target. (**a**) The spectrum of the received signal without clutter. (**b**) The spectrum of the received signal with clutter. (**c**) The spectrum after clutter suppression (FODS-SVD method). (**d**) The spectrum after clutter suppression (FEMP-SVD method). (**e**) Spectrum after clutter suppression (the proposed method).

**Figure 5:**
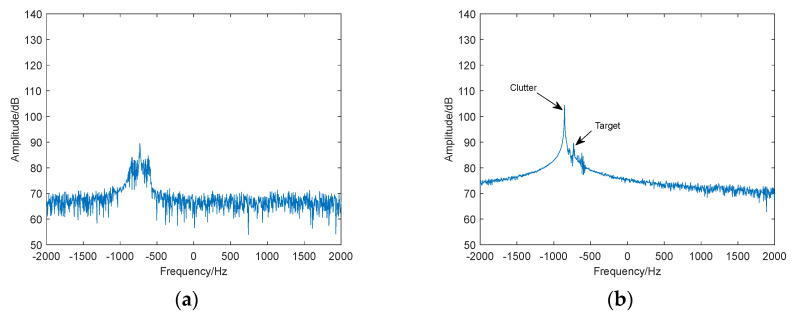

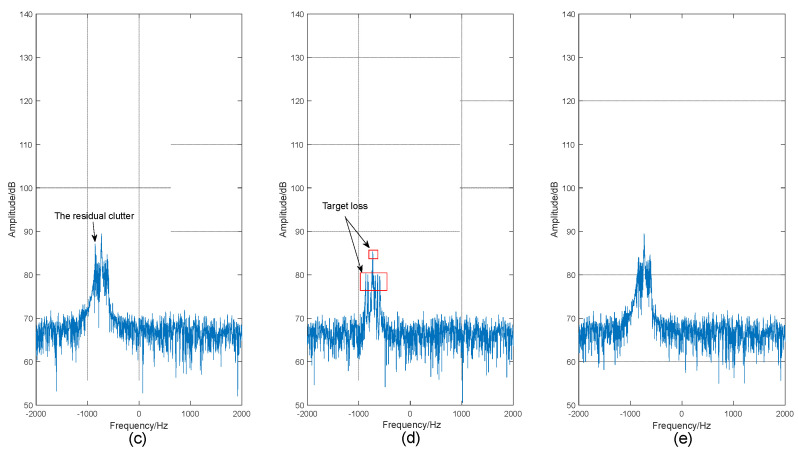
Clutter suppression results of a bird target. (**a**) The spectrum of the received signal without clutter. (**b**) The spectrum of the received signal with clutter. (**c**) The spectrum after clutter suppression (FODS-SVD method). (**d**) The spectrum after clutter suppression (FEMP-SVD method). (**e**) Spectrum after clutter suppression (the proposed method).

Based on the above parameters, by using the model that combines the movement characteristics of birds and the transmission characteristics of radar electromagnetic waves described in reference [36] and the scattering point superposition model described in reference [37], the spectrum of the received data without clutter is shown in Figure 4a and Figure 5a. The spectrum of the received data with clutter is shown in Figure 4b and Figure 5b. In the strong motion clutter environment (that is, the plane echoes), the target is essentially covered and cannot be detected. The results after clutter suppression are shown in Figure 4c–e and Figure 5c–e for the FODS-SVD method, the FEMP-SVD method, and the proposed method, respectively.

Compared to the results obtained by the FODS-SVD method, the proposed method successfully suppresses motion clutter, avoiding the existence of residual clutter caused by the situation where the singular values of clutter components are smaller than those of the target information. Compared to the results obtained by the FEMP-SVD method, the proposed method preserves relatively complete target information.

**Figure 6:**
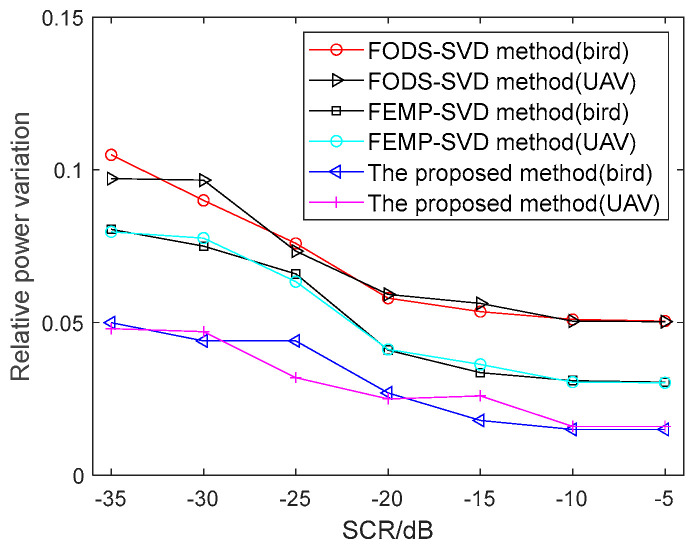
Relative power variation comparison after the clutter suppression.

To quantitatively evaluate the performance of the proposed method, the relative variance between the signal power after clutter suppression and the power of the received signal without clutter is defined [38] as follows:

(19) $$\mathrm{err}=\frac{{\left\|{y-y}_{r}\right\|}_{2}^{2}}{{\left\|y\right\|}_{2}^{2}}$$

where $y_{r}$ is the amplitude of the bird or UAV target without clutter and y is the signal amplitude of the bird or UAV after clutter suppression. The smaller the variance, the better the performance of the clutter suppression method. The relative power variations for the proposed method, the FODS-SVD method, and the FEMP-SVD method are shown in Figure 6. Using 200 sets of Monte Carlo experiments for each signal-to-clutter ratio, with each set containing 200 bird samples and 200 UAV samples, it can be observed that the relative power variance decreases and then tends to stabilize as the SCR increases.

The computational complexity of the proposed method is analyzed and compared with that of the FODS-SVD and FEMP-SVD methods, as shown in Table 4. The computer CPU model is Intel(R) Core (TM) i7-12700, and the main frequency is 2.10 GHz. It can be seen from Table 4 that the computation efficiency is lower compared with the FODS-SVD and FEMP-SVD methods. The proposed method performs two SVDs to ensure that the target is not lost and to suppress clutter as much as possible. However, this improved performance comes at the cost of increased computational complexity.

**Figure 7:**
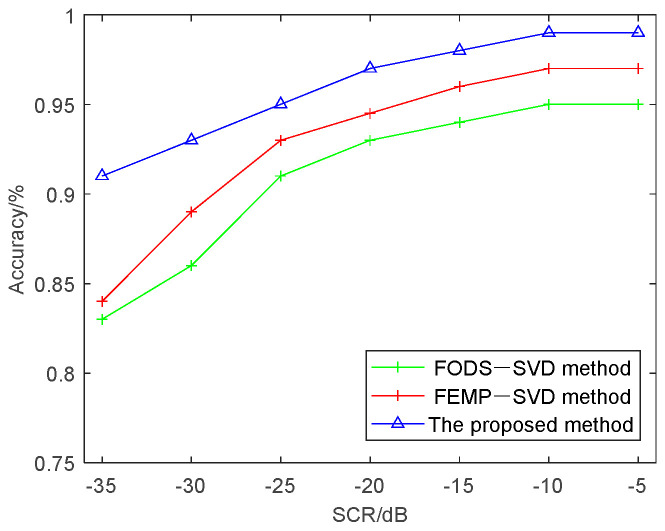
Performance comparison of the three methods.

After clutter suppression, the accuracy of target discrimination between birds and UAVs is depicted in Figure 7. As the signal-to-clutter ratio increases, the discrimination accuracy also improves. Compared to the FODS-SVD and FEMP-SVD methods, the proposed method significantly enhances discrimination accuracy in environments with strong motion clutter.

### 4.2. Results and Analysis of the Experimental Data

**Figure 8:**
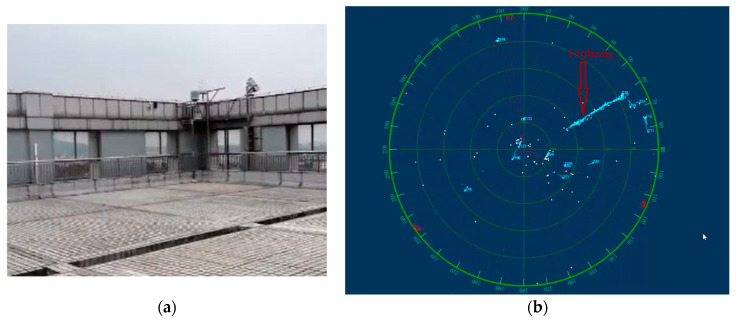
(**a**) The actual radar environment. (**b**) Radar beam pattern.

The radar operates in the C-band frequency range in the experimental data, with the clutter being primarily caused by car echoes from a nearby highway (as shown in Figure 8b). The actual radar data acquisition environment and radar beam pattern are shown in Figure 8a,b.

**Figure 9:**
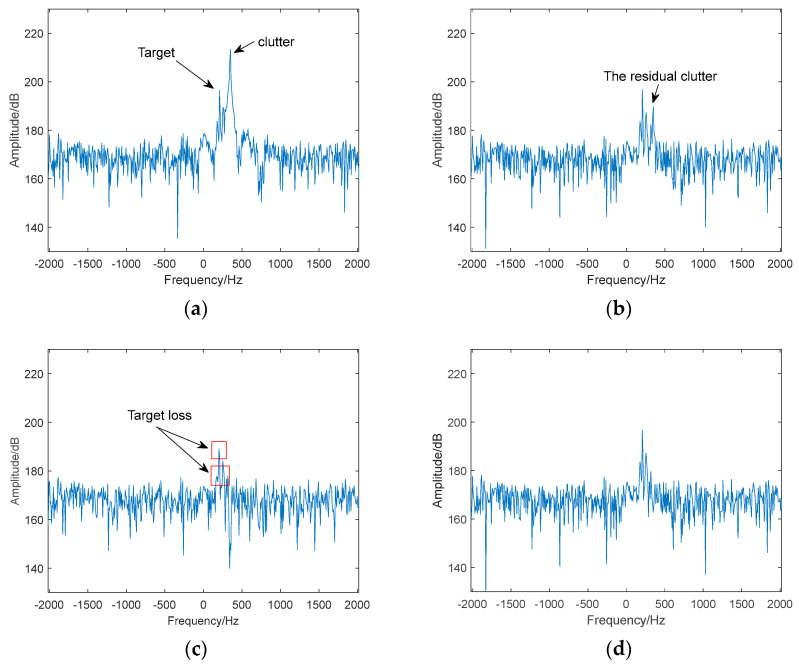
Clutter suppression results of the bird target. (**a**) The spectrum of the received signal. (**b**) Spectrum after clutter suppression (FODS-SVD method). (**c**) Spectrum after clutter suppression (FEMP-SVD method). (**d**) Spectrum after clutter suppression (the proposed method).

**Figure 10:**
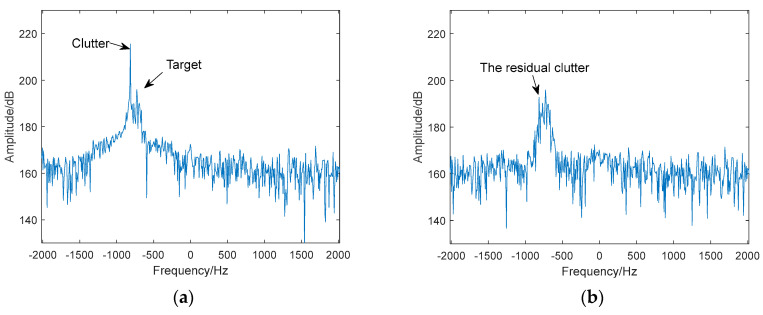

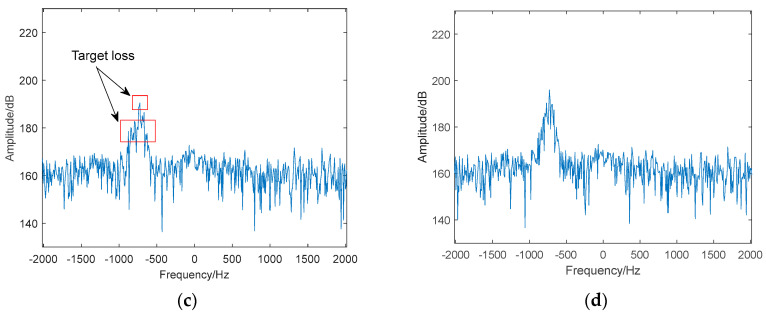
Clutter suppression results of the UAV target. (**a**) The spectrum of the received signal. (**b**) Spectrum after clutter suppression (FODS-SVD method). (**c**) Spectrum after clutter suppression (FEMP-SVD method). (**d**) Spectrum after clutter suppression (the proposed method).

The radar parameters include a pulse repetition time (PRT) of 62 µs, a fast sampling rate of 16 MHz, and a bandwidth of 8 MHz. The coherent processing interval (CPI) is 127 milliseconds, so the signal length N is 2048. The spectral correlation threshold is set to 0.8 and the spectral entropy threshold is set to 7 bits in the clutter suppression method. The received radar echoes of pigeons and DJI Phantom 4 UAVs, after ground clutter suppression, are shown in Figure 9a and Figure 10a, respectively. It is evident that strong motion clutter largely obscures the target echoes. The results of clutter suppression using the FODS-SVD, FEMP-SVD, and proposed methods are presented in Figure 9b–d and Figure 10b–d, respectively.

From Figure 9b–d and Figure 10b–d, compared to the results obtained by the FODS-SVD method, the proposed method successfully suppresses motion clutter, avoiding the existence of residual clutter caused by the situation where the singular values of clutter components are smaller than those of the target information. Compared to the results obtained by the FEMP-SVD method, the proposed method preserves relatively complete target information.

**Figure 11:**
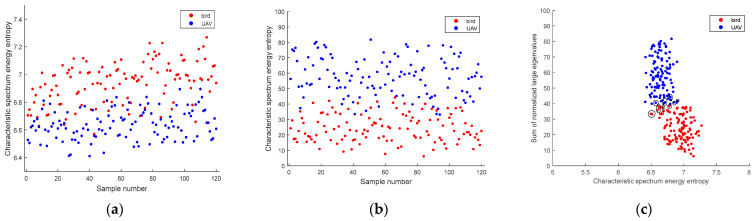
Identification results of birds and UAVs. (**a**) Characteristic spectral energy entropy. (**b**) Sum of normalized large eigenvalues. (**c**) Results obtained by kernel fuzzy c-means clustering.

The discrimination results between birds and UAVs are presented in Figure 11. Figure 11a,b display the extracted characteristic spectrum energy entropy and the sum of normalized large eigenvalues, respectively. After fusing these two features, the kernel fuzzy c-means method is employed to identify birds and UAVs, as illustrated in Figure 10c. The discrimination accuracy using the proposed clutter suppression methods is summarized in Table 5. Misjudged samples are indicated with circles in Figure 11c. For experimental data, our method enhances discrimination accuracy by 11.68% compared to the FODS-SVD method and by 6.28% compared to the FEMP-SVD method.

## 5. Conclusions

In this paper, we propose a frequency correlation dual-SVD reconstruction method to mitigate the impact of strong motion clutter on distinguishing between UAVs and birds. The proposed method preserves target micro-motion information while effectively suppressing clutter. We validate its effectiveness using both simulated and experimental data. Analysis of the simulation data reveals that the proposed method exhibits smaller relative power variation between the clutter-suppressed signal and the target signal (bird or UAV) without clutter, compared to the FODS-SVD method and the FEMP-SVD method. Our approach significantly improves the discrimination accuracy of birds and UAVs under low signal-to-clutter ratios. Specifically, for experimental data, the proposed method enhances discrimination accuracy by 11.68% compared to the FODS-SVD method and by 6.28% compared to the FEMP-SVD method.

## Figures and Tables

**Table 1:** Radar parameters.

Parameters	Value
Frequency	5.5 GHz
PRT	80 μs
Sampling Rate	80 MHz
Bandwidth	20 MHz

**Table 2:** Parameters of UAVs.

Parameters	Value
Number of rotors	4
Number of blades	2
Blade Length	120 mm
Rotating Rate	50 r/s, 64 r/s

**Table 3:** Parameters of birds.

Parameters	Value
Upper arm length	0.15 m
Forearm length	0.2 m
Bird body length	0.3 m
Tapping frequency	8 Hz

**Table 4:** The analysis of computational complexity.

Clutter Suppression Methods	Calculation Time
The FODS-SVD method	1.42 s
The FEMP-SVD method	1.88 s
The proposed method	2.98 s

**Table 5:** Identification accuracy of birds and UAVs.

Clutter Suppression Methods	Number of False Alarms	Total Sample Size	Identification Accuracy
The FODS-SVD method	44	240	81.7%
The FEMP-SVD method	31	240	87.1%
The proposed method	16	240	93.38%

## Data Availability

Data are contained within the article.
